# Distinct mutational landscapes and phylogenomic insights of the SARS-CoV-2 delta variant in Pakistan and India: Regional evolution, novel mutations, and epidemiological implications

**DOI:** 10.1371/journal.pone.0340704

**Published:** 2026-02-27

**Authors:** Nazia Fiaz, Atia Basheer, Imran Zahoor, Saima Naveed, Tahir Yaqub

**Affiliations:** 1 Genetic and Genomic Laboratory, Deptartment of Animal Breeding and Genetics, University of Veterinary and Animal Sciences, Lahore, Pakistan; 2 Department of Animal Nutrition, University of Veterinary and Animal Sciences, Lahore, Pakistan; 3 Institute of Microbiology, University of Veterinary and Animal Sciences, Lahore, Pakistan; Central Laboratory for Evaluation of Veterinary Biologics, Agricultural Research Center, EGYPT

## Abstract

Delta SARS-CoV-2 triggered a devastating wave of COVID-19 in India, infecting approximately 26% of the population (~357 million people) within four months, with ~0.4 million new cases per day, and around 250,000 reported deaths. In contrast, Pakistan experienced a much milder Delta wave, recording only 320,333 infections and 6,287 deaths. Against this epidemiological background, the current study aimed to identify genomic mutations in the delta-variant isolates reported from India and Pakistan and to compare their mutational profiles and phylogenomic patterns, without inferring direct clinical causality, in these neighboring countries. We analyzed 1,312 Pakistani and 3,140 Indian delta-variant genomes from the GISAID database to characterize their mutational spectrum and phylogenomic relationships in a global context. A total of 3,412 mutations were identified in Pakistani isolates compared with 6,856 mutations in Indian samples, reflecting differences in sample size, with NSP3 and spike protein emerging as the most frequently mutated regions. After normalization on a per-genome basis, Pakistani Delta genomes showed a slightly higher mutation density across most ORFs. Several globally common mutations, including ORF8: S84L, ORF1b: P1000L, and S: D157–158 deletions, were absent in both countries. Indian delta-genomes exhibited distinctive variants such as an M1M mutation in ORF7a and multiple stop-gain mutations in ORF3a, ORF7a, ORF8, ORF10, and NSP4, which may reflect population-specific evolutionary patterns rather than definitive effects on pathogenicity or transmissibility. In contrast, Pakistani isolates carried three novel missense mutations, NSP12b: Q348H, ORF6: K42E, and ORF3a: Y211H, at low prevalence. Phylogenomic analysis revealed that Pakistani isolates clustered primarily with Middle Eastern lineages, particularly from Saudi Arabia and Oman, suggesting international travel–linked introductions, rather than implying exclusive transmission routes. These findings highlight distinct regional evolutionary trajectories of delta-variant and demonstrate that differences in total mutation counts do not directly translate into per-genome mutational burden or disease severity. Our results emphasize the need for continued genomic surveillance to monitor region-specific viral adaptations and inform public health strategies.

## Introduction

The delta-variant (B.1.617.2) of SARS-CoV-2 was first detected in India on October 5, 2020 [1]. However, this variant quickly gained global attention because it was 40–60% more transmissible than alpha-variant [2], and 97% more transmissible and twice as infectious as the original Wuhan strain [3]. Clinical observations also indicated that patients suffering with delta-variant infections had 1.8-times higher risk of hospitalization compared with the alpha-variant infections [4]. According to some Chinese studies viral loads in delta-variant infections were approximately 1000-times higher than the viral loads in other variants infections [5,6]. Additionally, delta-variant showed evidence of escape from the immune response and antibody-based treatments [7,8]. Owing to these features, World Health Organization (WHO) declared delta as a variant-of-concern (VOC) on May 11, 2021 [1], and, shortly thereafter in June 2021, described it as “the fastest and fittest” SARS-CoV-2 variant due to its high transmissibility and disproportionate impact on the peoples suffering with chronic diseases like diabetes [9–11]. Moreover, it also contributed to the third wave of pandemic in many countries in Africa, Asia, Europe, North & South Americas [12] and became globally dominant variant as it continued to evolve and mutate [13,14]. However, in Pakistan the first confirmed patient of this variant was reported on May 16, 2021 and this lineage drove the country’s 4^th^ wave of COVID-19, beginning in mid-July 2021 [15].

Like other SARS-CoV-2 variants, Delta accumulated a distinct constellation of mutations across the Spike and non-structural proteins [16,17] that enhance its ACE2 affinity, viral entry, and immune evasion [18,19]. Notably, delta-variant harbor ten spike mutations, out of which L452R, T478K, and P681R were the characteristic Delta-mutations associated with enhanced transmissibility and reduced antibody neutralization [20–23]. The T478K mutation strengthen the RBD-ACE2 interaction and thus promote the cell entry [20,21], while P681R, located near the furin cleavage site, facilitate efficient membrane fusion and cell entry [24–26].

The Delta-variant drove India’s deadly second wave of COVID-19 beginning in February 2021 [27] during which daily cases averaged about 0.4 million [28] and about 26% (~357 million people) of the Indian population was infected over a four month period, and resulting in ~250,000 recorded deaths [12,29]. By contrast, in Pakistan’s delta-driven 4th wave, total number of cases and deaths were just 320,333 and 6,287, respectively [30]. To investigate potential genomic factors underlying these contrasting outcomes, we undertook a detailed mutational analysis of delta-variant populations of Pakistan and India by retrieving 1312 and 3140 high-quality, whole-genome sequences, respectively, from GISAID [31]. Hence, by tracking the delta-variant genomes, present study provides deep insight into the differences in the numbers and prevalence of mutations in Indian and Pakistani delta-populations, compared with its global pattern, and highlights the potential reasons why delta proved particularly severe in India. Finally, we performed phylogenomic analysis of delta-variant isolates from 14 different countries with substantial Pakistani diaspora and frequent travel links, which helped infer the probable routes of introduction of this variant to Pakistan.

## Materials and methods

### Genome retrieval and initial metadata processing

From the all publicly available SARS-CoV-2 Delta variant genomes (B.1.617.2) deposited from Pakistan and India on the GISAID EpiCoV™ database, a set of 1312 and 3140 whole genome sequences were retrieved along with their associated metadata for Pakistan and India respectively. Metadata included sample collection date, submission date, sequencing laboratory, and GISAID accession IDs. Notably, all genomes were collected at the end of the Delta wave, when both countries had completed submission of their Delta-lineage sequences to GISAID database. The Wuhan-Hu-1 genome (NC_045512.2) was used as the reference for genomic coordinate numbering and mutation identification throughout the analysis.

Because the majority of genomes from both Pakistan and India were submitted toward the end of the Delta wave, evenly distributed early-, mid-, and late-phase datasets were not available. Consequently, month-wise temporal trend analysis of mutation emergence was not performed, as such stratification would have introduced sampling bias.

### Quality control, filtering, and duplicate removal

To ensure reliability and comparability between Pakistani and Indian datasets, stringent and uniform quality-control (QC) criteria were applied to all genomes. Only sequences with a minimum length of 29,000 bp and fewer than 1% ambiguous bases (Ns) were retained. Genomes were required to be classified as “complete” and “high-coverage” according to GISAID standards. Sequences flagged for incomplete coverage, sequencing artefacts, or biologically implausible features such as frameshifts or premature stop codons (likely resulting from technical errors) were removed. Duplicate genomes were eliminated by comparing EPI_ISL accession identifiers as well as by computing SHA-256 sequence checksums to identify identical FASTA entries. Genomes lacking essential metadata, including sampling date, geographic location, or submitting laboratory, were excluded. After applying these QC filters uniformly to both countries, the final dataset consisted of 1,312 high-confidence Pakistani genomes and 3,140 high-confidence Indian genomes, all meeting consistent analytical standards.

### Rationale for the final sample size

To determine the final dataset used for comparative genomic analysis, all 1,312 Pakistani genomes that passed QC filtering were retained, as the total number of high-quality sequences available from Pakistan was relatively limited. In contrast, because over 100,000 Delta genomes were available from India, a sampling strategy was needed to avoid overrepresentation of particular states, laboratories, or outbreak clusters. Therefore, a time-stratified and region-stratified random sampling design was applied: all high-quality Indian genomes were first grouped by month of collection and by state/union territory, and proportionate random sampling without replacement was conducted within each stratum. This approach ensured that the reduced Indian dataset (n = 3,140) remained geographically and temporally representative, preserved lineage diversity, and minimized sampling bias, while maintaining sufficient statistical power to robustly compare mutation prevalence and evolutionary patterns between the two countries.

### Multiple sequence alignment

All quality-filtered SARS-CoV-2 genomes were aligned using MAFFT v7.480 [32,33], employing the high-accuracy L-INS-i algorithm optimized for full-length viral genomes. The alignment was performed using a gap-opening penalty of 1.53, a gap-extension penalty of 0.123 [34], and nucleotide mode settings, with the Wuhan-Hu-1 reference genome (NC_045512.2) included to standardize positional numbering across sequences. Following automated alignment, all FASTA files were visually inspected in MEGA-X [35] and BioEdit (v7.2.5) [36] to verify correct placement of indels, assess regions of high divergence, and confirm overall alignment quality prior to downstream mutation analysis.

### Mutation detection and annotation

Mutation profiling for each genome was performed relative to the Wuhan-Hu-1 reference sequence using the Coronapp web application [37], which identifies single-nucleotide substitutions, insertions, deletions, frameshift events, in-frame indels, and stop-gain mutations. To ensure analytical accuracy, Coronapp outputs were cross-validated using an in-house Python-based comparison of aligned FASTA files, and all discordant calls were manually reviewed in BioEdit and MEGA-X. Global prevalence estimates for key mutations were retrieved from Outbreak.info, enabling contextual comparison of Pakistani and Indian mutation patterns with worldwide Delta-variant trends.

### Statistical comparison of mutation prevalence

For each detected mutation, prevalence was calculated independently for Pakistani, Indian, and global Delta-genomes. Statistical differences between Pakistan and India were assessed using two-sided chi-square (χ²) tests with a significance threshold of p < 0.05. This approach allowed evaluation of whether specific mutations were significantly enriched in one population relative to the other, thereby enabling identification of country-specific mutation patterns with potential epidemiological relevance.

### Functional and protein-level annotation

All mutations were classified according to genomic location, including structural, nonstructural (NSP), and accessory ORFs and functional consequence, such as missense, synonymous, stop-gain, frameshift, or in-frame insertion/deletion. A heatmap summarizing mutation prevalence across Pakistan, India, and global datasets was generated to visualize regional differences in variant composition. Functional interpretation focused particularly on known Delta-defining spike mutations (e.g., L452R, T478K, P681R) as well as novel or low-frequency mutations unique to either country, integrating published SARS-CoV-2 protein annotations to infer potential biological impacts.

To account for differences in the number of genomes between countries, all mutation counts were normalized on a per-genome basis by dividing total mutations in each ORF by the number of high-quality genomes included per country (Pakistan: n = 1,312; India: n = 3,140). Normalized values are provided in Supplementary S1 Table.

### Global phylogenomic tree construction

To reconstruct robust evolutionary relationships among global SARS-CoV-2 Delta genomes, we assembled a geographically balanced dataset of 1,328 high-quality genomes obtained through stratified subsampling across all countries represented in the study. For countries with large genome availability, we selected 100 representative sequences from each of Australia, Canada, England, France, Germany, India, Italy, Norway, Oman, Pakistan, Saudi Arabia, the USA, and South Africa, and the, only available, 28 sequences from the United Arab Emirates. All genomes were aligned using MAFFT v7.505 (FFT-NS-2 strategy) with Wuhan-Hu-1 (NC_045512.2) as the reference. Maximum-likelihood phylogenetic inference was performed using IQ-TREE v2.1.4 under the GTR + G substitution model, with full model-parameter optimization and 1,000 ultrafast bootstrap replicates to assess branch support. The resulting ML tree was visualized in ITOL (https://itol.embl.de/).

## Results

**Fig 1:**
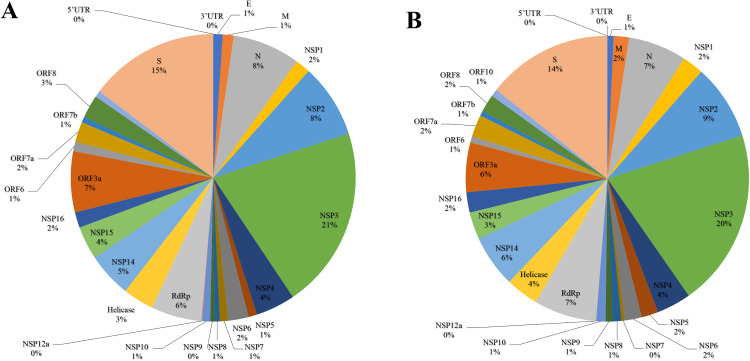
Pie-chart of mutation% observed in various proteins of the delta-variant samples submitted from Pakistan (A), and India (B).

A total of 3,412 mutations were identified in the Delta-variant genomes from Pakistan. These included 1,943 amino acid–changing mutations, 1,248 silent mutations, 123 non-coding region mutations, 2 in-frame deletions, 36 frameshift deletions, 1 in-frame insertion, 31 frameshift insertions, and 28 stop-gain mutations (Table 1; Fig 1A).

**Table 1:** Mutations found in various proteins of the delta-variant samples of Pakistan.

Protein	Missense	Silent SNP	Non-coding region		In-frame		Frameshift		Stop-gain	M1M	Total
			Mutation	Deletion	Deletion	Insertion	Deletion	Insertion			
E	21	13	–	–	1	–	–	3	1	–	39
M	24	39	–	–	–	–	–	–	–	–	63
N	149	77	–	–	–	–	2	2	2	–	232
NSP1	33	30	–	–	–	–	–	–	–	–	63
NSP2	162	78	–	–	–	–	2	1	1	–	244
NSP3	399	219	–	–	–	–	11	9	4	–	642
NSP4	87	61	–	–	–	–	1	1	–	–	150
NSP5	19	47	–	–	–	–	–	–	–	–	66
NSP6	45	43	–	–	–	–	–	–	–	–	88
NSP7	18	16	–	–	1	–	1	3	–	–	39
NSP8	12	14	–	–	–	–	–	–	–	–	26
NSP9	9	10	–	–	–	–	–	–	–	–	19
NSP10	18	24	–	–	–	–	–	–	–	–	42
NSP12a	2	1	–	–	–	–	–	–	–	–	3
RdRp	111	111	–	–	–	–	1	–	1	–	224
Helicase	68	72	–	–	–	–	2	1	–	–	143
NSP14	98	66	–	–	–	–	3	3	3	–	173
NSP15	73	44	–	–	–	–	–	–	–	–	117
NSP16	34	26	–	–	–	–	–	–	–	–	60
ORF3a	133	41	–	–	–	–	–	–	–	–	174
ORF6	21	10	–	–	–	–	–	–	1	–	32
ORF7a	44	20	–	–	–	–	–	–	6	–	70
ORF7b	12	5	–	–	–	–	–	–	1	–	18
ORF8	51	11	–	–	–	–	3	3	6	–	74
ORF10	14	9	–	–	–	–	–	–	–	–	23
S	286	161	–	–	–	1	10	5	2	–	465
5’UTR	–	–	44	–	–	–	–	–	–	–	44
3’UTR	–	–	79	–	–	–	–	–	–	–	79
**Total**	**1943**	**1248**	**123**	**0**	**2**	**1**	**36**	**31**	**28**	**–**	**3412**

In comparison, 6,856 mutations were detected in Delta genomes from India, nearly double the number observed in Pakistan. These consisted of 3,925 amino acid–changing mutations, 2,349 silent mutations, 229 non-coding mutations, 26 in-frame deletions, 122 frameshift deletions, 16 in-frame insertions, 42 frameshift insertions, 75 stop-gain mutations, and 72 M1M mutations (Table 2; Fig 1B). A bootstrap analysis (parametric Poisson model, 200,000 iterations) yielded a mutation-burden ratio of 2.01 (95% CI: 1.93–2.09), supporting the descriptive “nearly double” difference between the two datasets.

**Table 2:** Mutations found in various proteins of the delta-variant samples of India.

Protein	Missense	Silent SNP	Non-coding region		In-frame		Frameshift		Stop-gain	M1M	Total
			Mutation	Deletion	Deletion	Insertion	Deletion	Insertion			
E	28	22	–	–	–	–	–	1	–	–	51
M	69	72	–	–	–	–	1	2	1	–	146
N	261	151	–	–	2	1	6	1	2	–	424
NSP1	95	60	–	–	3	–	4	–	5	–	167
NSP2	336	169	–	–	–	–	3	3	5	–	516
NSP3	791	458	–	–	3	2	7	5	7	–	1273
NSP4	154	103	–	–	–	2	5	3	1	–	268
NSP5	75	65	–	–	–	–	2	–	–	–	142
NSP6	76	53	–	–	–	–	5	1	–	–	135
NSP7	15	24	–	–	–	–	–	–	1	–	40
NSP8	39	28	–	–	–	–	–	–	–	–	67
NSP9	31	20	–	–	–	–	2	–	–	–	53
NSP10	42	33	–	–	–	–	2	–	2	–	79
NSP12a	1		–	–	–	–	1	–	–	–	2
RdRp	280	224	–	–	–	–	7	1	1	–	513
Helicase	147	142	–	–	–	1	7	3	1	–	301
NSP14	233	135	–	–	2	2	8	3	8	–	391
NSP15	121	72	–	–	1	–	7	2	1	–	204
NSP16	90	64	–	–	–	1	–	–	3	–	158
ORF3a	220	72	–	–	1	–	4	–	4	–	301
ORF6	28	11	–	–	–	–	3	–	2	–	44
ORF7a	98	33	–	–	1	–	11	1	10	72	156
ORF7b	21	6	–	–	1	–	5	1	2	–	36
ORF8	79	20	–	–	6	–	4	–	10	–	119
ORF10	33	8	–	–	–	–	–	–	2	–	43
S	562	304	–	–	6	7	28	15	4	–	927
5’UTR	–	–	91	–	–	–	–	–	–	–	91
3’UTR	–	–	138	–	–	–	–	–	–	–	138
**Total**	**3925**	**2349**	**229**	**0**	**26**	**16**	**122**	**42**	**75**	**72**	**6856**

However, because the Indian dataset comprised a larger number of genomes, mutation counts were normalized on a per-genome basis. This analysis revealed that Pakistani Delta-genomes carried a slightly higher mutation density per genome across most ORFs compared with Indian genomes (Supplementary S1 Table).

### Protein-wise mutation distribution

To enable fair comparison between countries with unequal sample sizes, protein-wise mutation patterns are interpreted primarily using per-genome normalized mutation density (Supplementary S1 Table), while total mutation counts are reported descriptively.

Among the total 3,412 mutations detected in Pakistani genomes, 2,099 occurred within ORF1ab, which encodes the 16 non-structural proteins (NSPs). NSP3 contained the largest number of mutations (399 missense, 219 silent), followed by NSP2 (162 missense, 78 silent). The RNA-dependent RNA polymerase (RdRp) harbored 224 mutations, and ORF3a contained 174. ORF6, ORF7a, ORF7b, ORF8, and ORF10 showed 32, 70, 18, 74, and 23 mutations, respectively (Table 1).

Across structural proteins, the Spike (S) protein carried 465 mutations, including 286 missense mutations, 161 silent mutations, 1 in-frame insertion, 10 frameshift deletions, 5 frameshift insertions, and 2 stop-gain mutations. The nucleocapsid (N) protein contained 232 mutations, while the membrane (M) and envelope (E) proteins contained 63 and 39 mutations, respectively. The 5′UTR and 3′UTR regions harbored 44 and 79 mutations (Table 1).

### Prevalent mutations in Pakistan and India

Comparisons in this section are based on mutation prevalence (%) rather than absolute mutation counts, thereby minimizing the effect of unequal sample sizes between countries.

Several mutations such as NSP3: F106F, S: D614G, S: P681R, M: I82T, and ORF3a: S26L showed >99% prevalence in Pakistani samples, consistent with global frequencies (98–99%; Table 3). Some globally common missense mutations, including ORF1b: P1000L, ORF1b: ORF8: A1918V, ORF8: S84L, S: D157–158 deletions, were not detected in Pakistani sequences.

**Table 3:** Mutations in the delta-variant genomes submitted from Pakistan and India (prevalence ≥0.02).

Genomic change	Protein	Amino acid change	Type of mutation	Mutation% (Pakistan)	Mutation% (India)	Global%	χ²-value
19220C > T	NSP14	Missense	A394V	83.54	67.17	89.15	71.523***
11201A > G	NSP6	Missense	T77A	83.25	67.18	89.04	68.634***
10029C > T	NSP4	Missense	T492I	83.93	67.32	89.00	74.692***
9053G > T	NSP4	Missense	V167L	83.73	67.14	88.93	74.281***
4181G > T	NSP3	Missense	A488S	83.35	67.48	88.75	68.242***
6402C > T	NSP3	Missense	P1228L	82.1	67.64	87.14	55.844***
7124C > T	NSP3	Missense	P1469S	81.62	67.17	86.41	54.435***
21987G > A	S	Missense	G142D	67.37	45.22	66.5	100.158***
28915 CG > TT	N	Missense	G215C	83.45	67.99	65.3	64.463***
27874C > T	ORF7b	Missense	T40I	83.35	67.55	65.2	67.479***
21846C > T	S	Missense	T95I	59.38	50.45	33.5	16.357***
22227C > T	S	Missense	A222V	9.43	15.76	12	18.597***
27739C > T	ORF7a	Missense	L116F	4.91	11.50	11.4	28.934***
5184C > T	NSP3	Missense	P822L	15.4	29.55	9.09	57.818***
9891C > T	NSP4	Missense	A446V	14.92	27.39	8.81	46.847***
11418T > C	NSP6	Missense	V149A	14.92	27.58	8.65	48.191***
17236A > G	NSP13	Missense	I334V	2.41	0	7.13	24.291***
29427G > A	N	Missense	R385K	4.14	4.90	7.1	NS
11514C > T	NSP6	Missense	T181I	9.62	15.61	7.06	16.345***
1191C > T	NSP2	Missense	P129L	5.77	12.61	2.49	27.677***
25352G > T	S	Missense	V1264L	2.41	0	2.47	24.291***
28253C > A	ORF8	Missense	F120L	0	20.03	2.3	222.222***
21137A > G	NSP16	Missense	K160R	2.89	0	1.59	29.427***
27406C > T	ORF7a	Missense	L5F	0	2.04	1.44	20.202***
18176C > T	NSP14	Missense	P46L	0	6.34	1.36	65.049***
28249A > T	ORF8	Missense	D119V	0	3.63	1.2	222.222***
18255G > T	NSP14	Missense	M72I	16.27	0	1.11	177.463***
6408C > T	NSP3	Missense	S1230F	0	5.35	1.03	55.498***
24110A > C	S	Missense	I850L	2.21	0	0.89	22.245***
25439AG > CC	ORF3a	Missense	K16T	2.21	0	0.87	22.245***
25538G > T	ORF3a	Missense	G49V	16.84	5.41	0.76	65.850***
1268G > T	NSP2	Missense	D155Y	0	2.01	0.74	20.202***
26054C > A	ORF3a	Missense	T221K	16.94	5.48	0.63	65.335***
25667C > T	ORF3a	Missense	S92L.	0	4.27	0.6	43.945***
6539C > T	NSP3	Missense	H1274Y	4.43	2.17	0.60	7.584**
27389C > G	3’UTR	Extragenic	27389	0	2.04	0.58	20.202***
22899G > T	S	Missense	G446V	2.69	6.43	0.5	15.761***
25775G > T	ORF3a	Missense	W128L	0	7.23	0.5	74.689***
23012G > C	S	Missense	E484Q	3.27	8.82	0.5	26.610***
25740G > T	ORF3a	Missense	Q116H	2.5	0	0.5	25.316***
20320C > T	NSP15	Missense	H234Y	4.04	5.13	0.47	NS
25647G > T	ORF3a	Missense	L85F	0	10.73	0.44	113.048***
29868. > A	3’UTR	Extragenic	29868	0	2.96	0.43	30.457***
20396A > G	NSP15	Missense	K259R	0	2.01	0.43	20.202***
9203G > A	NSP4	Missense	D217N	0	2.04	0.41	20.202***
9678T > C	NSP4	Missense	F375S	0	2.04	0.41	20.202***
11083G > T	NSP6	Missense	L37F	2.12	2.77	0.4	NS
27390G > T	3’UTR	Extragenic	27390	8.76	0	0.40	92.05***
28248GATTTC > .	ORF8	Deletion	D119	0	31.56	84.1	375.297***
6573C > T	NSP3	Missense	S1285F	10.2	0	0.35	107.482***
27281G > T	ORF6	Missense	W27L	0	5.80	0.3	59.732***
25720G > T	ORF3a	Missense	A110S	2.31	0	0.3	23.268***
29862G > A	3’UTR	Extragenic	29862	0	2.23	0.30	22.245***
29690G > T	3’UTR	Extragenic	29690	2.79	0	0.30	28.398***
20578G > T	NSP15	Missense	V320L	3.18	0	0.22	32.520***
17964G > T	NSP13	Missense	M576I	2.12	0	0.21	21.223***
28703G > C	N	Missense	D144H	0	2.23	0.2	22.245***
18106G > T	NSP14	Missense	A23S	0	4.20	0.2	42.901***
27494C > T	ORF7a	Missense	P34L	2.12	0	0.2	21.223***
14120C > T	NSP12b	Missense	P218L	3.08	0	0.2	31.488***
4683C > T	NSP3	Missense	A655V	0	2.23	0.13	22.245***
25166G > C	S	Missense	E1202Q	2.98	0	0.12	30.457***
20480C > T	NSP15	Missense	S287L	2.31	0	0.11	22.245***
16741G > T	NSP13	Missense	V169F	2.6	0	0.11	26.342***
22027. > G	S	Insertion	S155	0	8.60	0.10	89.864***
6828C > T	NSP3	Missense	S1370F	18	0	0.053	197.802***
6120C > T	NSP3	Missense	S1134L	0	2.26	0.05	23.268***
3542A > G	NSP3	Missense	T275A	0	4.14	0.04	41.858***
19687C > T	NSP15	Missense	P23S	0	2.20	0.04	22.245***
23058C > G	S	Missense	P499R	0	3.85	0.04	38.736***
8915T > C	NSP4	Missense	F121L	2.12	0	0.01	21.223***
28271A > .	3’UTR	Extragenic	28271	0	4.08	0	Unique (India)
27915G > T	ORF8	Stop-gain	G8*	0	2.77	0	Unique (India)
27762G > T	ORF7b	Stop-gain	E3*	0	4.08	0	Unique (India)
25522G > T	ORF3a	Stop-gain	G44*	0	6.97	0	Unique (India)
27514G > T	ORF7a	Stop-gain	E41*	0	7.99	0	Unique (India)
9559C > A	NSP4	Stop-gain	Y335*	0	3.73	0	Unique (India)
16703A > .	NSP13	Deletion	E156	0	33.69	0	Unique (India)
28273A > .	3’UTR	Extragenic	28273	0	41.24	0	Unique (India)
45G > T	5’UTR	Extragenic	45	2.02	0	0	Unique (Pak)
38A > C	5’UTR	Extragenic	38	2.69	0	0	Unique (Pak)
196A > C	5’UTR	Extragenic	196	2.79	0	0	Unique (Pak)
187A > C	5’UTR	Extragenic	187	4.72	0	0	Unique (Pak)
27758G > A	ORF7a	M1M	*122*	0	72.55	0	Unique (India)
26023T > C	ORF3a	Missense	Y211H	2.21	0	0	Unique (Pak)
27325A > G	ORF6	Missense	K42E	2.69	0	0	Unique (Pak)
14511G > C	NSP12b	Missense	Q348H	8.66	0	0	Unique (Pak)
29567AT>TG	ORF10	SNP-stop	I4*	0	5.00	0	Unique (India)

The M1M mutation in ORF7a was observed at 72.55% prevalence in India but was absent in Pakistan and the global dataset. Similarly, the S: E156G mutation showed extremely low prevalence in Pakistan (0.19%) compared with 86.7% globally. India also exhibited multiple stop-gain mutations (e.g., E41* in ORF7a; G44* in ORF3a; Y335* in NSP4; G8* in ORF8) with low but notable prevalence. These were not observed in Pakistani or global datasets (Table 3).

Additional mutations, such as F120L, L85F, W128L, P46L, W27L, and others were present at moderate frequencies in Indian samples (5–33%) but absent in Pakistani sequences. Conversely, the D119–120 deletion in ORF8, common globally (84.1%), showed low prevalence in both datasets (0.096% in Pakistan; 31.56% in India). Several mutations including NSP3: P822L, NSP4: A446V, NSP6: V149A, S: A222V, and others appeared at substantially higher prevalence in Indian sequences, whereas G49V, N72I, T221K, S1285F and S1370F had higher prevalence in Pakistan than globally, with limited representation in Indian data.

Mutation counts were normalized per genome. Pakistan showed 2.60 mutations/genome, whereas India showed 2.18 mutations/genome (Supplementary S1 Table). Per-protein normalization demonstrated that Pakistan had slightly higher per-genome mutation density across most ORFs (e.g., NSP3: 0.49 vs 0.41; Spike: 0.35 vs 0.30). Thus, the higher total number of mutations detected in India primarily reflects the larger number of genomes analyzed rather than a consistently higher per-genome mutational burden.

**Fig 2:**
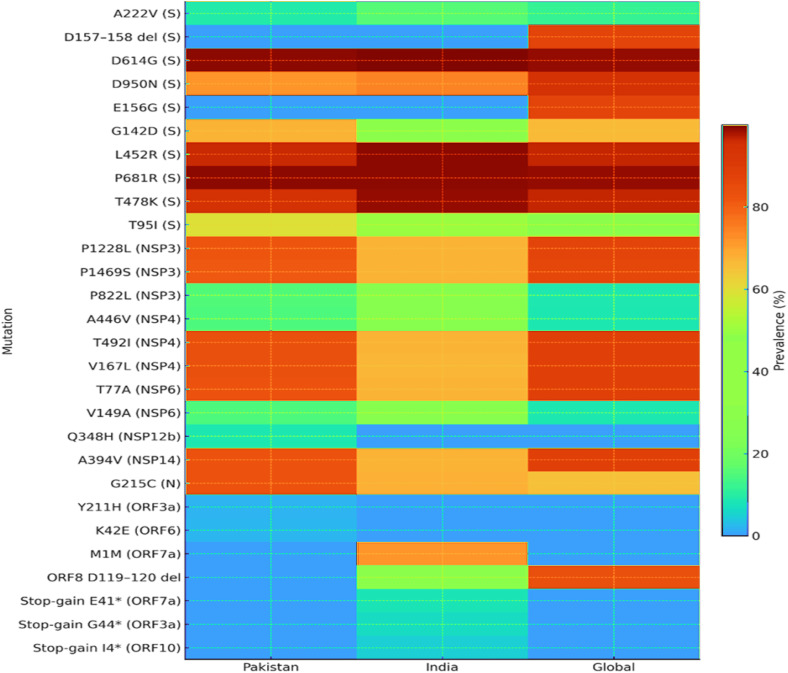
Heatmap of major Delta-variant mutations in Pakistani, Indian, and global SARS-CoV-2 genomes.

Overall, the heatmap (Fig 2) summarizes shared versus population-specific mutations and contrasts their prevalence against global frequencies.

### Phylogenomic analysis

**Fig 3:**
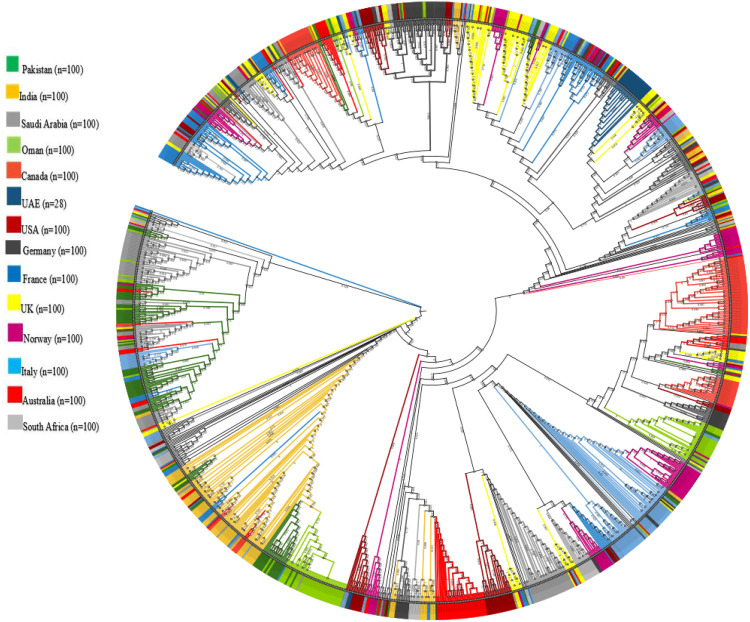
Phylogenomic tree of delta-variant sequences (n = 1328) from fourteen countries including Australia, Canada, France, Germany, Italy, Oman, Saudi Arabia, South Africa, UAE, UK, USA, Norway, India, and Pakistan.

The results of phylogenomic analysis (Fig 3) showed that Pakistani samples were grouped in many clusters showing their relationships with samples submitted from different countries. However, the closest relationship of Pakistani samples was observed with samples submitted from Saudi Arabia, followed by Oman, France, Norway, England, Australia, South Africa, India, and the United States. Interestingly, no relationship of Pakistani samples was found with the samples originated from Canada, Germany, and Italy, though a large number of Pakistani diasporas reside there.

## Discussion

This study provides a comparative framework for understanding how the SARS-CoV-2 Delta variant diversified across two closely connected yet epidemiologically distinct regions (Pakistan and India). Rather than merely documenting mutational differences, our analysis highlights how region-specific evolutionary trajectories, founder effects, and transmission networks may have contributed to the divergent genomic landscapes observed in the two countries.

A key finding of our work is the substantially higher total number of detected mutations in Indian Delta-genomes compared with Pakistani genomes. However, because total mutation counts are influenced by sample size, comparisons were further evaluated using per-genome normalized mutation density. Although mutation counts alone do not infer functional impact, the greater genomic diversity observed in India at a population level is consistent with reports that large, rapidly expanding epidemics promote accelerated intrahost evolution and the emergence of additional sublineages [38–40]. Such patterns have been documented globally in settings of high transmission intensity, where increased opportunities for replication increase the likelihood of new substitutions and indels [26,41,42]. In contrast, Pakistan’s smaller epidemic size may have constrained evolutionary space, resulting in fewer accumulated changes at the aggregate level [30]. This interpretation aligns with prior genomic surveillance studies from South Asia showing heterogeneous rates of Delta diversification shaped by demographic and epidemiological conditions [2,10,43].

Across both datasets, NSP3 and Spike remained the principal mutational hotspots, a hallmark of Delta evolution globally [40,44]. However, country-specific differences in NSP3, NSP4, and NSP6 substitutions suggest that distinct selective pressures or founder events may have acted within each population. For example, India showed elevated frequencies of NSP3: P822L, NSP4: A446V, and NSP6:V149A, all of which have been associated, experimentally or computationally, with altered membrane remodeling or replication dynamics [45–47]. Pakistan, on the other hand, displayed higher prevalence of variants such as T492I and V167L in NSP4, which align more closely with globally predominant patterns [48]. Importantly, these contrasts reflect differences in mutation prevalence rather than per-genome mutation rates, underscoring that even shared variants of concern can accumulate geographically structured accessory mutations, a pattern widely reported for Delta-sublineages in Europe, Asia, and South America [23,39,49–52]. Recent genomic surveillance studies published after 2023 have further shown that Delta diversification frequently involved region-specific circulation of AY sublineages, including AY.4 and AY.122, highlighting continued heterogeneity in Delta evolutionary trajectories across geographic settings [39–41,51].

Spike N-terminal domain differences further illustrate this divergence. Mutations such as E156G and the S155 insertion, frequently detected in India, were rare or absent in Pakistan, echoing earlier findings that these NTD changes emerged preferentially in regions with intense transmission and immune pressure [53,54]. Meanwhile, the absence of globally dominant D157–158 deletions in both countries suggests that South Asian Delta genomes followed trajectories distinct from those dominant in Europe and the Americas during the same period [1,13].

Differences also extended to accessory genes. India exhibited multiple ORF3a, ORF7a/b, and ORF8 stop-gain or disruptive mutations, whereas Pakistan did not. Previous studies have suggested these truncations may modulate innate immune antagonism or apoptosis pathways, though their exact phenotypic effects remain variable and context-dependent [26,55,56]. Importantly, our results do not infer clinical or epidemiological impact; rather, these patterns reflect distinct evolutionary experiments occurring across viral populations exposed to different epidemiological and immunological environments.

Non-coding region variation also differed substantially. The 3′UTR 28273A> variant was common in India but absent in Pakistan. This variant and similar 3′UTR polymorphisms has been associated with changes in RNA stability and replication efficiency in recent structural studies [18,57]. These findings emphasize the need to incorporate regulatory-region variation into future functional analyses, which often focus disproportionately on Spike.

Taken together, these results demonstrate that although Pakistan and India share the canonical Delta genomic backbone, they harbor distinct sets of accessory and non-structural mutations. Such divergence reflects the broader principle that variants of concern do not evolve uniformly across regions; instead, they branch into geographically structured sublineages influenced by population immunity, demographic conditions, movement patterns, and stochastic founder events [10,53,58].

Phylogenomic analysis further contextualizes these findings. Pakistani Delta genomes clustered more closely with isolates from Saudi Arabia, Oman, and several European countries than with those from India, despite geographic proximity. This pattern suggests, but does not definitively establish, diaspora-driven mobility supersedes regional adjacency in shaping introduction pathways [59]. The lack of strong linkage to Indian sequences suggests that Pakistan’s Delta outbreaks were seeded primarily through Middle Eastern travel corridors, highlighting the importance of integrating genomic surveillance with international mobility data [15,60,61]. Such insights carry practical implications for border-screening and travel-policy design.

Overall, our findings underscore the complexity of SARS-CoV-2 evolution in densely connected regions and emphasize the need for sustained genomic monitoring. While sequence-based analyses cannot establish causality regarding transmission dynamics or disease severity, they reveal evolutionary signatures that, when combined with clinical and demographic data, can enhance our understanding of variant behavior.

Because the analyzed genomes were predominantly submitted toward the end of the Delta wave, this study could not evaluate month-wise temporal trends in mutation emergence, which should be addressed in future analyses using longitudinally balanced datasets. In addition, this study did not perform site-specific recombination or selection pressure analyses (e.g., FUBAR, MEME, or FEL), as the primary focus was on comparative mutational profiling and phylogenomic structure; such approaches represent important avenues for future work aimed at resolving adaptive and selective mechanisms.

Future work should incorporate functional assays, immunological profiling, and travel-network modeling to clarify how accessory and non-structural mutations contribute to regional viral adaptation and to better anticipate the trajectories of emerging lineages.

## Conclusion

Taken together, our findings underscore the profound impact of regional mutational diversity on epidemic outcomes. In India, a higher mutational burden, the enrichment of immune-evading spike alterations, and the presence of multiple stop-gain mutations across accessory proteins likely enhanced transmissibility, immune escape, and pathogenicity. By contrast, the Pakistani delta population, characterized by fewer mutations, the absence of disruptive variants, and introduction via Middle Eastern rather than Indian routes, might have led to comparatively milder epidemic outcomes. The identification of novel Pakistani mutations further emphasizes that even regions experiencing milder epidemics can harbor unique variants with the potential to expand under selective pressure. This reinforces the critical need for continuous genomic surveillance, not only to monitor global variants of concern but also to detect emerging region-specific mutations that may alter the trajectory of future waves.

## Supporting information

S1 Table Normalized mutation burden per genome. (DOCX)
