# Supplementary material for: Distinct mutational landscapes and phylogenomic insights of the SARS-CoV-2 delta variant in Pakistan and India: Regional evolution, novel mutations, and epidemiological implications
Source: PLoS One. 2026 Feb 27;21(2):e0340704. doi: 10.1371/journal.pone.0340704 (PMC12948076; doi:10.1371/journal.pone.0340704)
Supplement: S1 Table — (DOCX) [file pone.0340704.s001.docx]

# **Table S1. Normalized Mutation Burden per Genome**

| **Protein** | **Pakistan (Mut/genome)** | **India (Mut/genome)** |
| --- | --- | --- |
| E | 0.0297 | 0.0162 |
| M | 0.0480 | 0.0465 |
| N | 0.1768 | 0.1350 |
| NSP1 | 0.0480 | 0.0532 |
| NSP2 | 0.1861 | 0.1643 |
| NSP3 | 0.4891 | 0.4054 |
| NSP4 | 0.1143 | 0.0854 |
| NSP5 | 0.0503 | 0.0452 |
| NSP6 | 0.0671 | 0.0430 |
| NSP7 | 0.0297 | 0.0127 |
| NSP8 | 0.0198 | 0.0213 |
| NSP9 | 0.0145 | 0.0169 |
| NSP10 | 0.0320 | 0.0252 |
| NSP12a | 0.0023 | 0.0006 |
| RdRp | 0.1710 | 0.1634 |
| Helicase | 0.1090 | 0.0958 |
| NSP14 | 0.1319 | 0.1245 |
| NSP15 | 0.0892 | 0.0649 |
| NSP16 | 0.0457 | 0.0503 |
| ORF3a | 0.1326 | 0.0958 |
| ORF6 | 0.0244 | 0.0140 |
| ORF7a | 0.0534 | 0.0497 |
| ORF7b | 0.0137 | 0.0114 |
| ORF8 | 0.0564 | 0.0379 |
| ORF10 | 0.0175 | 0.0137 |
| S (Spike) | 0.3544 | 0.2955 |
| 5’UTR | 0.0335 | 0.0290 |
| 3’UTR | 0.0602 | 0.0439 |
